# Visualization of house-entry behaviour of malaria mosquitoes

**DOI:** 10.1186/s12936-016-1293-7

**Published:** 2016-04-25

**Authors:** Jeroen Spitzen, Teun Koelewijn, W. Richard Mukabana, Willem Takken

**Affiliations:** Laboratory of Entomology, Wageningen University, PO Box 8031, 6700 EH Wageningen, The Netherlands; International Centre of Insect Physiology and Ecology (icipe), P.O. Box 30772-00100, Nairobi, Kenya; School of Biological Sciences, University of Nairobi, P.O. Box 30197 GPO, Nairobi, Kenya

**Keywords:** *Anopheles gambiae*, Bed net, Behaviour, Mosquito flight, House-entry, Video-recording, Semi-field, Kenya

## Abstract

**Background:**

Malaria mosquitoes often blood feed indoors on human hosts. The mosquitoes predominantly enter houses via open eaves. Host-seeking is odour-driven, and finding a host depends on the quality of the odour plume and whether the route towards the host is free of obstructions. Little is known about in-flight behaviour of mosquitoes during house entry. This semi-field study visualizes mosquito house entry in three dimensions (3D) and offers new insights for optimizing vector control interventions.

**Methods:**

The approach and house entry of *Anopheles gambiae* sensu stricto was studied in a semi-field set-up using video-recorded flight tracks and 3D analysis. Behavioural parameters of host-seeking female mosquitoes were visualized with respect to their position relative to the eave as well as whether a mosquito would enter or not. Host odour was standardized using an attractive synthetic blend in addition to CO_2_. The study was conducted in western Kenya at the Thomas Odhiambo Campus of the International Centre of Insect Physiology and Ecology, Mbita.

**Results:**

The majority of host-seeking *An. gambiae* approached a house with a flight altitude at eave level, arriving within a horizontal arc of 180°. Fifty-five per cent of mosquitoes approaching a house did not enter or made multiple attempts before passing through the eave. During approach, mosquitoes greatly reduced their speed and the flight paths became more convoluted. As a result, mosquitoes that passed through the eave spent more than 80 % of the observed time within 30 cm of the eave. Mosquitoes that exited the eave departed at eave level and followed the edge of the roof (12.5 %) or quickly re-entered after exiting (9.6 %).

**Conclusions:**

The study shows that host-seeking mosquitoes, when entering a house, approach the eave in a wide angle to the house at eave level. Less than 25 % of approaching mosquitoes entered the house without interruption, whereas 12.5 % of mosquitoes that had entered left the house again within the time of observation. Advances in tracking techniques open a new array of questions that can now be answered to improve household interventions that combat malaria transmission.

**Electronic supplementary material:**

The online version of this article (doi:10.1186/s12936-016-1293-7) contains supplementary material, which is available to authorized users.

## Background

The anthropophilic and historically endophilic malaria vector *Anopheles gambiae s.s.* is known to exploit host odours to find a suitable blood meal for egg production [1–3]. In a field setting, this would mean that after emergence as adults, initial nectar feeding and joining mating swarms, the mosquitoes enter into a host-seeking state which will commonly lead them to the vicinity of or into human dwellings [4, 5].

Navigation of the mosquitoes towards inhabited houses is initiated by increases in CO_2_ levels in combination with a number of host odours [6–8]. Odour plumes become wider and more diluted at greater distance from the source [9]. The turbulent forces of diffusion in wind stretches and stirs odour filaments, creating gaps of odour-free air in a plume while it expands and is transported with the wind [10]. Filaments are stirred around in eddies (ranging from millimetres to hundreds of metres) and intermingled with the surrounding odour-free air [9]. These turbulent forces in air cause a spatially complex and temporally changing three-dimensional distribution of odour concentrations in natural habitats [9]. Many insects exhibit behaviour that enables them to follow odour plumes to the source [10], however, there is much diversity in how insects navigate along such plumes to reach their target. For mosquitoes, one can expect differences between diurnal and nocturnal species and the multimodal use of visual cues or the relative importance of a general host cue such as CO_2_ [3, 11].

### House entry

Studies on mosquito house entry often describe the approach and entry of indoor-biting mosquitoes based on catches with traps that can intercept house entry or exit behaviours [5, 12, 13]. These studies are based on the knowledge that anopheline mosquitoes predominantly enter and exit via eaves [14, 15]. An extensive field study on *An. gambiae* on house entry is well described by Snow [14], who elaborates on earlier work in The Gambia reviewed by Gillies [16], in which the long-range orientation to human dwellings is attributed to the dispersion of increased CO_2_ levels. More recent work has looked at this topic from the point of house entry and has begun to unravel the role of specific host cues once mosquitoes are inside a house [17].

Grieco et al. [12] observed that doors, windows and eaves are large portals of house entry for *An. vestitipennis*. Host-seeking mosquitoes find little or no obstacle to entering a house when one of these openings is available [18]. Walls form obstacles, but some mosquitoes will still be able to enter a house if there are gaps in the wall ranging from 1–2 cm wide [12, 18]. Reviewing mosquito house entry, Lindsay et al. [19] concluded that houses with open eaves or houses that lack ceilings had higher numbers of mosquitoes and higher levels of malaria compared to neighbouring houses with closed eaves or ceilings. Snow suggests that the majority of mosquitoes fly below a height of 1 m towards a house and when they reach the house they will at some point fly upwards to eave level and then move indoors.

### Vector control interventions

Innovative control strategies are needed to reduce vector-host interactions for the control of malaria [20]. The dispersion of host cues is related to house design and this has, together with physical barriers, implications for the level of indoor biting [21–23]. Indirectly, the level of house entry also has an impact on the proportion of mosquitoes that would eventually try to feed outdoors, and may be responsible for outdoor malaria transmission [24, 25]. Along with the development of new tools, proper implementation is highly relevant: Jawara et al. [26] showed that odour-baited MM-X traps set 15 cm from the ground at 2 and 10 m from a house caught more mosquitoes than traps set at 150 cm from the ground, but next to the house comparable numbers of mosquitoes were caught at 15 and at 150 cm in height. The efficiency of push–pull strategies and other large-scale efforts to reduce parasite transmission can benefit from detailed behavioural descriptions on how repellents and attractants function spatially [27, 28].

Advances in tracking techniques can provide more detail on flight behaviours without artificial obstacles, such as interception traps, which are likely to affect initial flight paths [11, 29, 30]. The data provide new insights on how to disrupt mosquitoes’ natural behaviour or cause interference along their route [22, 23, 27, 31–33]. The present study describes the house entry and exit behaviour of mosquitoes that were lured towards a house by standardized human host cues in a semi-field setting. Three-dimensional (3D) reconstructions of recorded flight tracks were made, in which mosquitoes were observed to enter, exit or move away from the eave of an experimental house. By providing knowledge on flight behaviour of mosquitoes in situ, improvements of trapping systems, push–pull systems and netting/screening designs can be made.

## Methods

### Mosquitoes

*Anopheles gambiae* sensu stricto (Mbita strain) mosquitoes were obtained from the insectaries located at the Thomas Odhiambo Campus of the International Centre of Insect Physiology and Ecology (*icipe*-TOC) in Mbita Point township, western Kenya (00°25′S, 34°13′E). The Mbita strain has been reared since 2001 under ambient climatic conditions. Eggs were laid on wet filter paper and placed in filtered water from Lake Victoria, in plastic trays. Aquatic stages of the mosquitoes were reared in a screen house and larvae were fed daily on cat food (Go-Cat complete Purina, Croydon, England). Pupae were collected daily and transferred to mesh-covered (30 × 30 × 30 cm) cages. Mosquitoes used for the experiment did not have access to a blood meal. Mosquitoes were fed on a 6 % glucose (Excel Chemicals Ltd., Nairobi, Kenya) solution.

### Experimental set-up

**Fig. 1:**
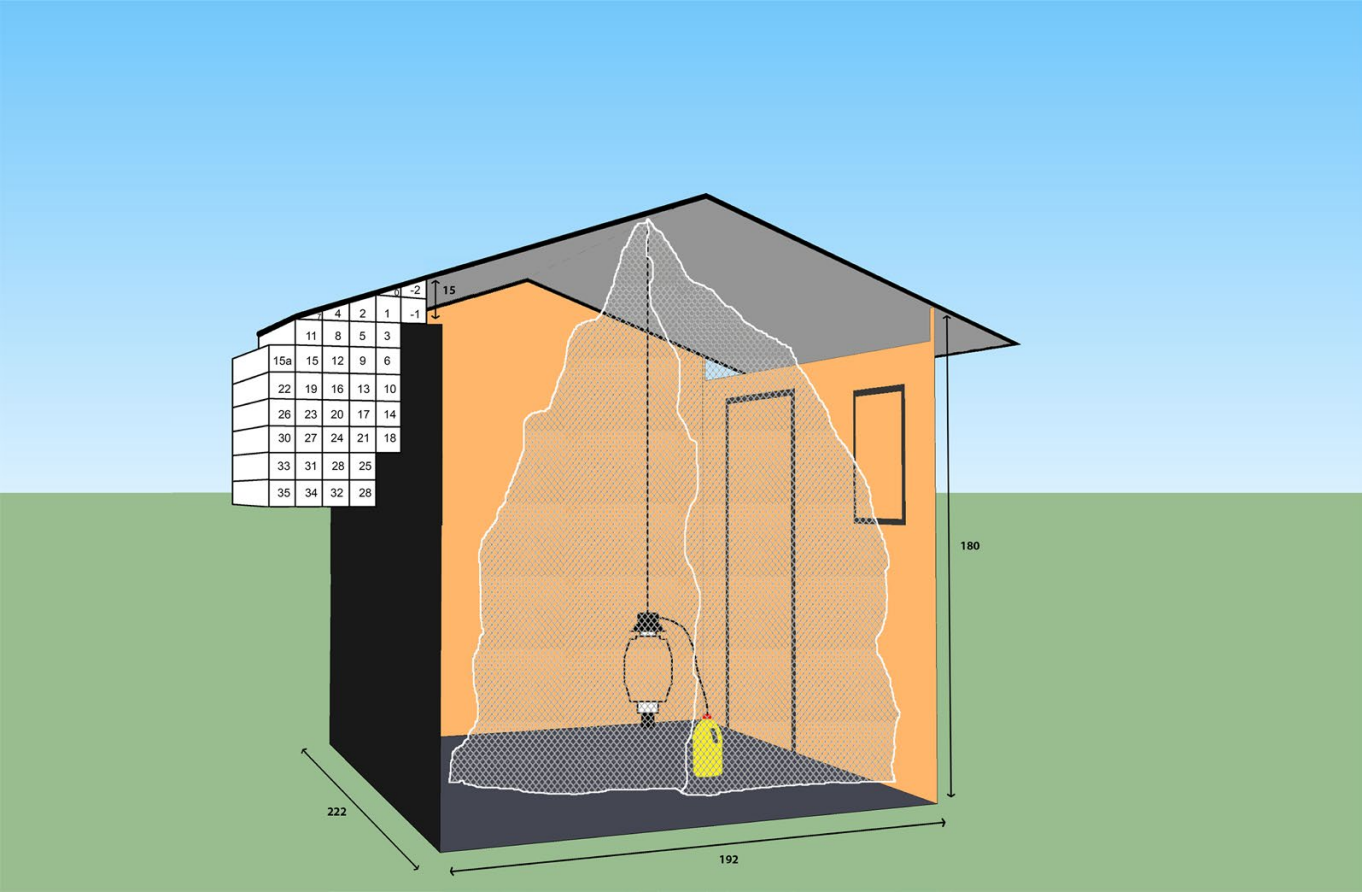
Experimental house with tracked area superimposed. Measurements of the house and eave in cm with block-shaped zones of 70 × 10 × 10 cm for which 3D-analysis of flight parameters were performed. An untreated net was hung over an odour-baited MM-X trap during the course of filming

Experiments were done inside a screen house, the semi-field set-up, located at *icipe*-TOC. The screen house (flight arena) was made of wooden poles and beams and a roof of thatch covered with corrugated iron. The flight arena of the mosquitoes was 12.8 × 4.75 × 2.25 m. The screen house was covered with dark-green shade netting permitting airflow (wind) to enter the system. Inside the house there was another netting to prevent the escape of any released mosquitoes. The base of the screen house consisted of a top layer of sand and pebble-sand and an under layer of rocks and compact sand. An experimental house, constructed of wood and a corrugated iron roof, was built inside the screen house. The house had two windows and a door. In many African houses there is a gap between the top of the wall and the roof, an open eave, to provide ventilation. The experimental house had a 15-cm wide eave on two sides of the house. Instead of filming the full distance across one eave, the camera view covered the central area in front of the house and below the eave to obtain an as large as possible tracking arena with 3D coverage (Fig. 1). Parts of the wall and roof were covered with black recycled polycarbonate (WSV Kunststoffen, Utrecht, The Netherlands) to optimize the contrast during filming. Inside the experimental house, there was a hook for suspending a Mosquito Magnet^®^ X (MM-X) odour-baited trap and for a bed net. The release point of the mosquitoes was at 4.25 m in front of the experimental house. Attached to the screen house, a control room of 5.40 × 1.80 m was installed where a computer and all other operating systems were placed. There were no persons around in the screen house or the control room during the recording phase.

### Recording equipment

Two Cohu 4722-2000/0000 monochrome CCD camera (Cohu, San Diego, CA, USA) equipped with Fuji non-tv 9 mm/f1.4 lenses were installed on a solid frame dug into the sand at 35 cm from the house facing the eave. Four Tracksys (Nottingham, UK) infrared light units were placed just behind the cameras, each unit contained an array of 90 infrared LEDs emitting light with peak output at 880 nm. The equipment was powered via an uninterruptible power supply (UPS) and computer controlled in the control room. Details on the automated tracking system and 3D reconstruction are based on the description given by Spitzen et al., [30]. A lightweight calibration frame was constructed of pieces of Cochranes Orbit molecular building system (Cochranes of Oxford Ltd, UK) containing 71 markers. The calibration accuracy of the set-up was 0.23 % of the dimensions of the tracking arena.

### Experimental procedures

The MM-X trap was used to dispense synthetic host odours that lured mosquitoes into the experimental house and to remove experimental mosquitoes after the behavioural recordings had taken place. An untreated bed net (100 % polyester, Top 2, Kenya) was hung over the trap during the flight experiment. The composition, production and dispersion of the five-component blend and CO_2_ is described in detail by [34] and [35] with the exception of tetradecanoic acid (C14) that was used in a 0.0025 g/L concentration. Female mosquitoes were three to eight days old when tested and were aspirated from the adult cages eight to 10 h before the start of the experiment. They were placed in plastic cups of 1 L (PCL Mombasa, Kenya) in groups of 200 covered with mosquito gauze and provided with a paper towel soaked in water.

The trap started running at 20.00 h, from the moment mosquitoes were released. Mosquitoes were released remotely, by lifting the lid of the mosquito container using a fishing line. At 00.00 h, the cameras were stopped and the trap was uncovered from the bed net allowing trap entry and mosquito removal from the experimental cage. At 10.30, the MMX-trap was stopped and placed in a freezer. After 15 min the blend was removed from the trap and stored in a refrigerator at 4 °C. Caught mosquitoes were counted.

### Data processing and analyses

3D output data were filtered for mosquitoes with a minimum of six recorded frames (0.24 s) while flying. A filter for flying was set at >27 mm/sec, being the speed of the fastest walking mosquito observed, and speeds above 2000 mm/sec were left out as this removes most artefacts that were taken as target. In the rare event where >one mosquito came in view at the same time, only the mosquito that was tracked first was used for the analysis and the other(s) ignored.

Data were sub-divided into four groups depending on where a mosquito came into view of both cameras and was last seen in the tracking arena. These groups were referred to as: 1: in view, not entering eave; 2: in view, entering eave; 3: exiting and re-entering eave; 4: exiting eave and departing. As individual mosquitoes could get out of view, it is possible that they were observed more than once in the same or different groups within the 4 h of recording.

To analyse the spatial pattern of flight behaviours, the 3D space in front of the eave was divided in block-shaped zones of 70 × 10 × 10 cm (see Fig. 1). The percentage of time flying per zone was first calculated for each mosquito and averaged over all mosquitoes per group and plotted in heat maps. Flight speed was defined as the magnitude of the velocity vector in space and expressed in mm/sec. The angular change of heading was defined as the change of track angle for one time step, expressed in degrees. These two parameters were also averaged per zone block, with a minimum of six different contributing tracks per zone.

The data were not normally distributed. Differences between groups were tested for their significance using Kruskal–Wallis tests via SPSS statistics version 22 (IBM Corp, USA) followed by pair-wise multiple comparisons. Adjusted p values were calculated and considered significant at a threshold of p < 0.05.

## Results

### Flight behaviour around the eave

**Fig. 2:**
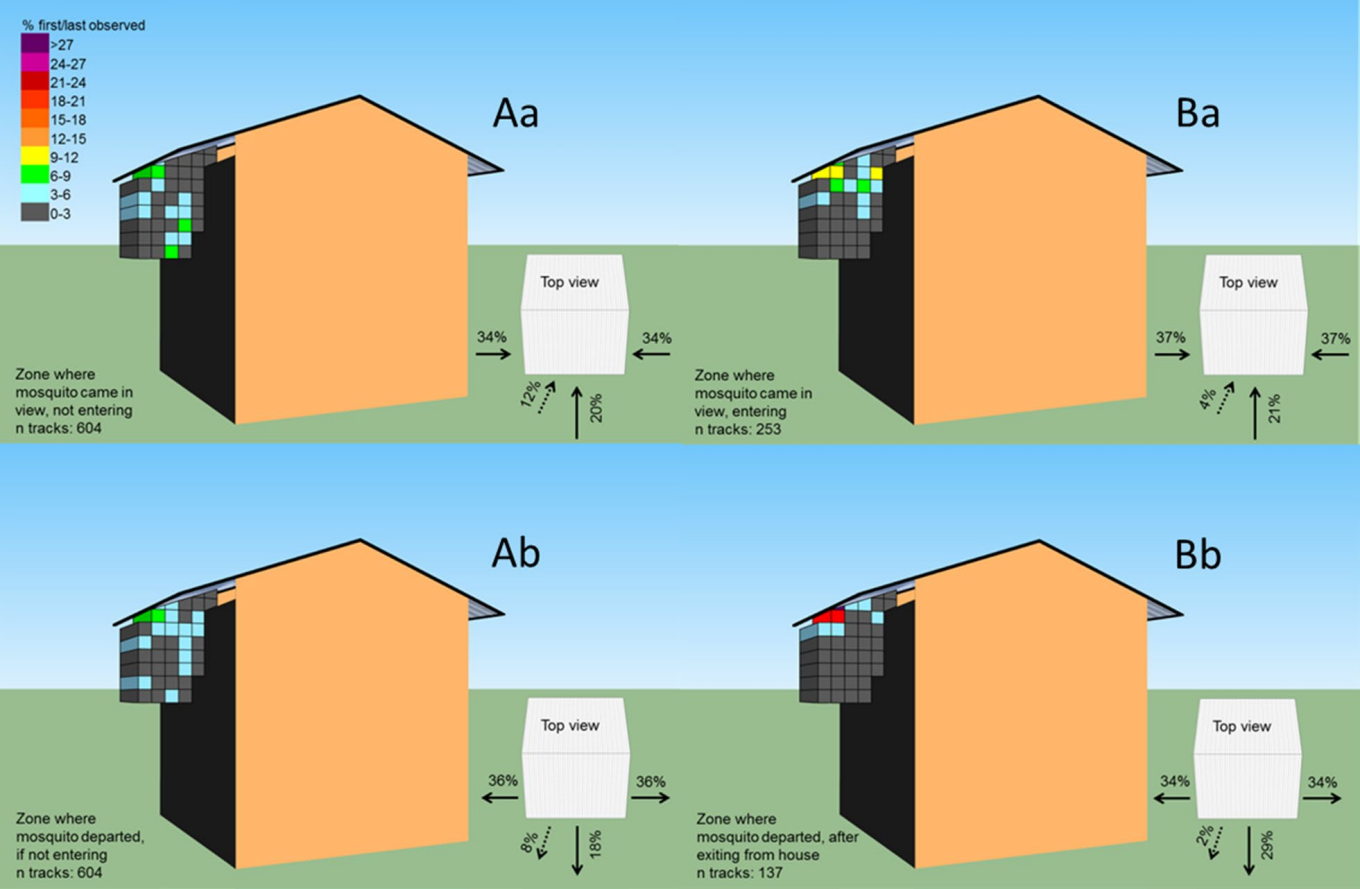
Observation arena where mosquitoes entered and left. The heat maps represent the frequency distribution in which zones mosquitoes were first or last observed within a reconstructed track. **Aa** and **Ab** represent mosquitoes that did not pass through the eave. **Ba** and **Bb** represent mosquitoes that passed through the eave. *Top view images* summarize the direction in which mosquitoes entered or left the area around the house

During five experimental nights, a total of 1000 mosquitoes were released, and 473 were caught in the MM-X trap, which they could enter after the bed net was folded (from 00.00 h onwards). Mosquitoes could get in view of the cameras multiple times, resulting in a total of 1100 tracks that were analysed with a minimum of six frames (0.24 s). Of the mosquitoes that were not observed to enter via the eave (n = 604), 67.5 % came first into view via zones categorized as ‘sides’, 19.9 % from the ‘front’, 11.9 % from ‘below’, and 0.7 % from below/front of the house. A selection of representative video images and track reconstruction is available via Additional file 1. A heat map representing the frequency distribution of first entries per zone is presented in Fig. 2Aa. This group of tracked mosquitoes left the filmed area without entering the house in similar proportions to how they entered the arena: 72.8 % from the sides, 18.2 % from the front, 7.9 % from below, and 1 % left from below/front (Fig. 2Ab). Of the 253 mosquitoes that were observed to enter the eave (excluding individuals that exited the eave and directly re-entered), 74.7 % came into view via the sides, 20.9 % from the front and 4.4 % were first tracked in the lowest zones (Fig. 2Ba). A large proportion (48.9 %) of the mosquitoes that exited the eave (n = 137) departed via the two zones just near the roof edge (Fig. 2Bb). When using the same categories as before, exiting mosquitoes were observed to depart for 68.6 % via the sides, 29.2 % from the front and 1.5 % from below (see top view panel Fig. 2Bb).

The maximum track duration ranged from 12 s for a mosquito that came into view and entered via the eave to 6.7 s for a mosquito that exited the house and departed. Most flight tracks were much shorter and largely dependent on where a mosquito would come from or where it was flying towards. The mean track duration of mosquitoes that came into view and entered via the eave was significantly longer than that of the group that came into view but did not enter (2.23 vs 1.51 s, Kruskal–Wallis, pair-wise comparison, p < 0.05, see Table 1). Mosquitoes that exited and re-entered the house remained in close proximity to the eave and had on average a significantly shorter track duration compared to all other groups (0.89 s, Kruskal–Wallis, pair-wise comparison, p < 0.05, see Table 1). Detailed information on the mean time spent in each zone at increasing distance from the eave is given in Additional file 2.

**Table 1:** Mean (±s.e.m.) track duration, speed and angular change of heading based on individuals with a minimum of six recorded frames (0.24 s)

Destination when in view	N (total 1100)	%	Mean track duration (s)	Mean flight speed (mm/sec)	Mean angle change 3D (degrees)
Did not enter house	604	54.9	1.51 ± 0.05 a	443.5 ± 5.4 a	12.3 ± 0.3 a
Entered house via eave	253	23.0	2.23 ± 0.09 b	378.8 ± 5.6 b	14.6 ± 0.5 b
Exited house and re-entered	106	9.6	0.89 ± 0.10 c	291.2 ± 12.0 c	25.5 ± 1.6 c
Exited house and departed	137	12.5	1.75 ± 0.09 b	412.1 ± 9.9 a	15.5 ± 0.8 b

Different letters within each column indicate significant differences in parameters depending on where mosquitoes entered/exited the filmed area (Kruskal–Wallis, pair-wise comparisons, adj. significance; p < 0.05)

**Fig. 3:**
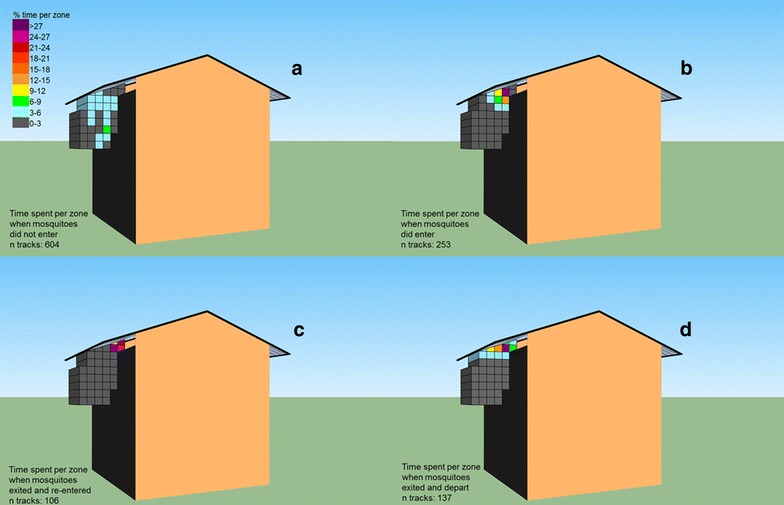
The spatial–temporal distribution per mosquito. *Colours* represent the percentage of time per zone for tracks in which mosquitoes were observed to **a** not pass the eave, **b** pass the eave, **c** re-enter the eave after exiting, **d** exit and leaving the filmed area

The spatial–temporal distribution of each individual flight path was calculated. The mean time spent per zone is plotted in heat maps and presented separately based on where mosquitoes where observed to leave the filmed area (Fig. 3ad). With a random distribution, one may expect that mosquitoes spent 2.6 % in each zone (100/38). Mosquitoes that did not enter the eave showed little variation in time spent between the zones, with a maximum of 6.9 % spent in one of the lower zones close to the wall (n tracks 604, Fig. 3a). This is in contrast to mosquitoes that were observed to enter the arena: 49.6 % of the time was spent in three zones close to the eave and 82.8 % within the 11 zones closest to the eave (n tracks 253, Fig. 3b). Mosquitoes that were observed to exit and re-enter the eave did not move far away and spent 95.0 % of the time within four zones close to the eave (n tracks 106, Fig. 3c). Departing mosquitoes show a gradient in time spent close to the eave, moving away from the eave and following the roof. In total 92.1 % depart and disappear from view via these upper zones (n tracks 137, Fig. 3d).

**Fig. 4:**
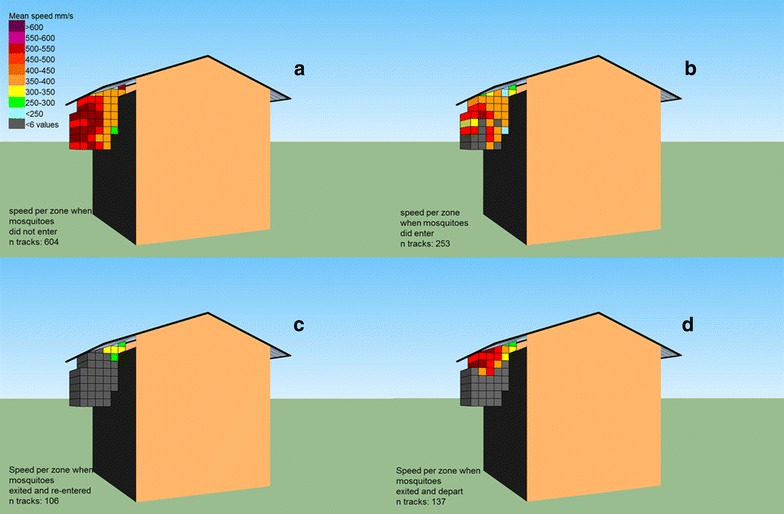
Mean flight speeds per zone. The mean flight speed per zone was analysed based on where mosquitoes entered or left the arena with a minimum of six different tracks per zone. **a** Not pass the eave, **b** pass the eave, **c** re-enter the eave after exiting, **d** exit and leaving the filmed area

Mosquito flight speed and the angular change of heading were analysed separately, depending on where a flying mosquito would leave the filmed area around the house. The mean flight speed over an entire track was lower for mosquitoes that came into view and entered the eave compared to mosquitoes that did not enter, or the ones that exited and departed (379 vs 444 and 412 mm/sec, respectively; Kruskal–Wallis, pair-wise comparisons, p < 0.05, see Table 1). Lowest flight speeds were recorded for mosquitoes that exited and re-entered the house with on average 291 mm/sec, (Kruskal–Wallis, pair-wise comparisons, p < 0.05, see Table 1). Figure 4a–d visualizes the mean speeds per zone at different distances from the eave, averaged per mosquito with a minimum of six data points (= the mean is calculated over a minimum of six mosquitoes) per zone and shows a general pattern in which speeds diminished by nearly 50 % when flying closer to the eave. This pattern is visible for both entering and exiting mosquitoes (Fig. 4b, d).

**Fig. 5:**
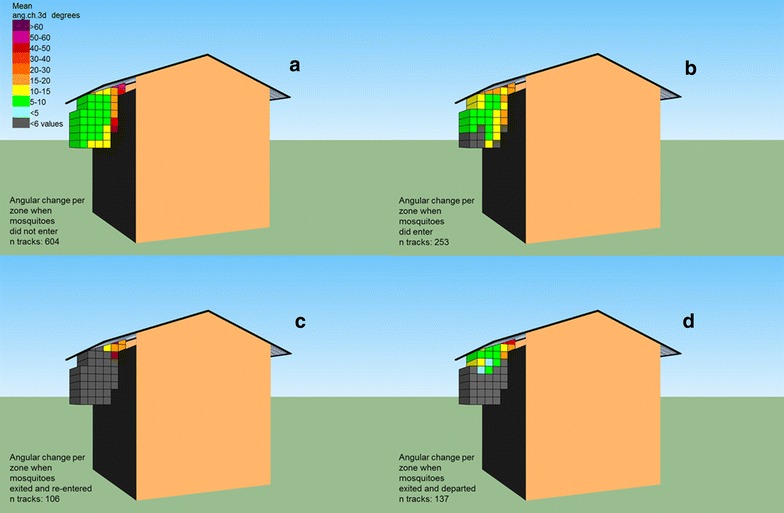
Mean angular change in 3D per zone measured in degrees.* Higher values* indicate a more convoluted flight path. The means per zone were analysed based on where mosquitoes entered or left the arena with a minimum of six different tracks per zone.** a** Not pass the eave, **b** pass the eave, **c** re-enter the eave after exiting, **d** exit and leaving the filmed area

The directness of flight is presented in Fig. 5a–d and shows higher convoluted tracks in zones close to the eave and near the wall or roof of the house. As a result, the mean angular change in 3D over the entire track was highest for mosquitoes that exited and re-entered the house. The mean values did not differ between mosquitoes that came into view and entered compared to mosquitoes that exited and departed (Kruskal–Wallis, pair-wise comparisons, p > 0.05, see Table 1).

## Discussion

This study describes the house entry and exiting behaviour of mosquitoes that were not intercepted by entry/exit traps and not impeded by physical obstacles when passing through the eave. Flight trajectories and their related parameters were different depending on whether mosquitoes were entering, exiting or moving away from a house. However, for all distinct groups, the majority of the mosquitoes that entered or exited the trackable area did this at eave level and spent just a few seconds there. There were no clear indications that mosquitoes arrived at low altitude and moved upwards along the wall towards the eave. This occurred most likely outside the range of the cameras, a few metres away from the house as suggested by Snow [14]. More information was gathered than in studies with interception traps, as with the filming techniques used in this study it was possible to distinguish the flight paths of mosquitoes that entered the house and of those that did not enter and moved away. One can only speculate on the successive behaviour of mosquitoes that did not enter. They could either return into view and eventually enter or move towards other openings that were not filmed and a proportion would not have been in a physiological state of host seeking [36, 37]. For those not host seeking, the endophilic nature of this malaria vector may still have caused movements towards the house and it would require individual tracking of mosquitoes in order to study whether this group would use different cues and/or routes to locate and enter a house [14, 17].

The standardized synthetic lure used did not include a heat source, however it proved to be able to lure mosquitoes into houses as if they were occupied by human beings, as shown in previous studies [38, 39]. Heat was considered as a relevant host cue with respect to mosquito landing and of lesser value for house entry, as it has been shown that at a distance >15 cm the effect of heat is no longer evident [30, 40]. Although there were no plants growing inside the screen house, the mosquitoes flew in an environment surrounded by plants and occupied buildings were present within 100 m. In other words, there were many competing olfactory cues that had the potential to interfere with a mosquito flight path towards the experimental house.

Mosquitoes that were associated with eave entry spent most of their time within proximity of the eave. Although there was a free passageway to fly through the eave, flight speed decreased nevertheless. This can be explained by short collisions with the walls or ceiling and/or part of the exploration strategy as earlier observed for *An. gambiae* during their response to host cues [30] and recently supported in a study on flight behaviour near bed nets [33]. The same arguments seem valid for the increase in angular change while getting close to the eave. These observations may not seem surprising in themselves, but they raise questions on how this behaviour would change in the presence of disrupting odours [27] or if eaves are (partly) blocked [21, 22]. Therefore, in field situations, what would be the giving-up time of host-seeking mosquitoes and how long would a mosquito be in contact with treated (netting) material impregnated with fungicides or insecticides [33, 41–43].

Previous studies showed that the presence of (competing) human volunteers inside an experimental house did not affect the results of odour-baited outdoor traps catches [26]. During the present study, odours were released inside the house, similar to those emanating from a human, and it would be interesting to find out, using advanced tracking techniques, at what altitude mosquitoes fly further away from the house as a detailed follow-up study by Gillies and Wilkes [44] and Snow [14]. As mentioned by several authors, optimal trapping height and thereby other interference tools too, vary between mosquito species and trap design [26, 28, 44].

House-exiting mosquitoes seemed to follow the roof edge with increasing speed and a more direct flight while moving away from the house. The authors are not aware of other data on free-flying mosquitoes that have described such behaviour. Mosquitoes that left the house had not obtained a blood meal and were likely to move on in search of other host cues.

House design and its geographical positioning influences air currents and odour plume structures [6, 21]. This study presents the results of a house in a semi-field setting, however, recent developments in filming techniques will make it possible to upscale the sample size and to conduct tests in more rural settings. High-speed cameras with increased resolution will allow filming of a larger sample area where fast-moving multiple insects can be tracked [33]; (M Lankheet and JS unpublished data). Ideally, next-generation tracking systems are interlinked with ambient weather data in order to correct for prevailing airflows on the actual airspeed and flight direction of the mosquito. Further developments in tracking techniques could reduce the need for specially constructed experimental houses and enhance knowledge of free-flying mosquitoes [15, 45] to improve vector intervention tools.

## Conclusion

The malaria mosquito *An. gambiae s.s*. approaches houses at eave level in a wide, near-horizontal arc. More than half of the examined mosquitoes that approached the house did not proceed to enter the eave. The obtained knowledge on house entry behaviour can be exploited for the development of intervention tools that focus on the disruption of vectors that enter human-occupied buildings. Such interventions should focus on interferences at eave level, with the knowledge that *An. gambiae s.s.* often passes this route in only a few seconds.

## Additional files

10.1186/s12936-016-1293-7 Animation of the experimental set-up including a selection of representative video images and tracking results.
10.1186/s12936-016-1293-7 Mean time spent per zone. Means calculated over the total time per zone of each individual with a total track length of six frames (0.24 s). n tracks represent the number of times a zone was visited by different mosquitoes. The solid fills highlight the value in the cell. A longer bar represent a higher value.
