# Supplementary material for: Visualization of house-entry behaviour of malaria mosquitoes
Source: Malar J. 2016 Apr 25;15:233. doi: 10.1186/s12936-016-1293-7 (PMC4843208; doi:10.1186/s12936-016-1293-7)
Supplement: Supplementary file 2 — 10.1186/s12936-016-1293-7 Mean time spent per zone. Means calculated over the total time per zone of each individual with a total track length of six frames (0.24 s). n tracks represent the number of times a zone was visited by different mosquitoes. The solid fills highlight the value in the cell. A longer bar represent a higher value. [file 12936_2016_1293_MOESM2_ESM.docx]

**Additional file 2 Mean time spent per zone**

Means calculated over the total time per zone of each individual with a total track length of six frames (0.24 sec). n tracks represent the number of times a zone was visited by different mosquitoes. The solid fills highlight the value in the cell. A longer bar represent a higher value.

Zone

Mean (s)

n tracks

Zone

Mean (s)

n tracks

Zone

Mean (s)

n tracks

Zone

Mean (s)

n tracks

-2

0.10

6

-2

0.18

58

-2

0.21

50

-2

0.17

51

-1

0.17

7

-1

0.12

107

-1

0.18

84

-1

0.17

83

0

0.19

9

0

0.21

78

0

0.19

49

0

0.18

76

1

0.58

34

1

0.49

248

1

0.58

71

1

0.46

136

2

0.57

70

2

0.48

130

2

0.61

8

2

0.31

113

3

0.58

106

3

0.44

162

3

0.61

11

3

0.51

25

4

0.51

91

4

0.58

59

4

1.08

3

4

0.25

84

5

0.32

107

5

0.28

126

5

0.16

2

5

0.26

34

6

0.51

115

6

0.37

75

6

0.64

2

6

0.33

5

7

0.38

66

7

0.52

32

7

0.40

3

7

0.26

55

8

0.28

107

8

0.31

72

8

0.30

2

8

0.27

47

9

0.32

127

9

0.23

97

10

0.04

1

9

0.30

11

10

0.39

94

10

0.31

36

11

0.12

2

10

0.18

2

11

0.29

111

11

0.25

48

11

0.19

36

12

0.23

120

12

0.21

58

12

0.24

22

13

0.30

154

13

0.23

53

13

0.32

4

14

0.27

76

14

0.17

18

14

0.16

2

15

0.26

117

15

0.22

40

15

0.21

24

16

0.25

111

16

0.20

40

16

0.27

8

17

0.34

145

17

0.22

32

17

0.66

2

18

0.13

32

18

0.09

6

18

0.08

1

19

0.25

99

19

0.13

28

19

0.19

9

20

0.22

100

20

0.20

17

20

0.22

5

21

0.42

136

21

0.39

22

21

0.88

2

22

0.11

46

22

0.06

13

22

0.31

4

23

0.23

96

23

0.23

12

23

0.14

2

24

0.21

93

24

0.14

11

24

0.24

4

25

0.28

94

25

0.45

11

25

0.28

2

26

0.15

49

26

0.11

6

26

0.13

3

27

0.22

57

27

0.22

5

27

0.04

1

28

0.21

104

28

0.15

13

28

0.21

3

29

0.13

36

29

0.09

3

30

0.20

1

30

0.17

45

30

0.12

8

32

0.36

2

31

0.19

46

31

0.16

2

32

0.28

75

32

0.44

8

33

0.19

40

33

0.76

1

34

0.15

30

34

0.09

3

35

0.13

10

35

0.12

2

**Exit and Depart**

**Arrive and Enter**

**Exit and Re-enter**

**Arrive Not Enter**
